# Predictive Factors of the Entrepreneurial Performance of Undergraduates

**DOI:** 10.3389/fpsyg.2022.814759

**Published:** 2022-03-11

**Authors:** Qu Shaowei, Li Tianhua, Zhang Miao

**Affiliations:** ^1^Institute of Educational Economics and Management, University of Science and Technology Beijing, Beijing, China; ^2^School of Economics and Management, University of Science and Technology Beijing (USTB), Beijing, China; ^3^School of Humanities and Social Science, University of Science and Technology Beijing, Beijing, China

**Keywords:** college entrepreneurs, entrepreneurial performance, structural equation model, predictive factors, entrepreneur’s personality

## Abstract

College students have gradually become the main force of entrepreneurship in mass entrepreneurship and innovation. However, their entrepreneurial performance was not as good as expected. We have carried out research to analyze the predictive factors of entrepreneurial performance of college students and put forward targeted suggestions, hoping to be helpful to improve their entrepreneurial performance of them. Based on questionnaire data obtained from 2,097 college student entrepreneurs, this study uses the structural equation model to analyze the predictive factors of the entrepreneurial performance of college students. The survey results of the questionnaire show that both personal and behavioral factors influence the entrepreneurial performance of college students. In this study, personal factors in this study mainly include entrepreneurial willingness, personality, and ability of entrepreneurs. Behavioral factors mainly refer to the positive behaviors of entrepreneurs that can affect entrepreneurial performance.

## Introduction

In recent years, affected by various factors such as the epidemic situation and the expansion of enrollment, the employment circumstances of college students have become increasingly severe, and the problem of employment difficulties has become increasingly prominent. The employment quality of college students has become a key concern in society. College students’ innovative thinking is active and novelty. Their entrepreneurial activities contribute to alleviating employment pressure and stimulating economic growth. The stimulation of economic vitality through undergraduate entrepreneurship is a meaningful and realistic topic. China is in a transitional period of economic development. Innovation and entrepreneurship will surely become crucial to promoting the country’s future economic quality.

However, the success rate of undergraduates in China is still low. For improving the entrepreneurial qualities of undergraduates, the first thing we need to do is analyze the influencing factors of their entrepreneurial performance. This research aims to investigate the predictive factors of the entrepreneurial performance of undergraduates, construct a structural equation model, and propose corresponding training paths based on the model. The study of the entrepreneurial performance of college students is conducive to promoting the vigorous development of entrepreneurial activities of college students, alleviating the problem of “difficult employment,” and promoting the high-quality transformation of the Chinese economy.

## Literature Review

### Definition of Undergraduate Entrepreneurship

The mainstream concept of entrepreneurship in China generally asserts that entrepreneurship is a process during which people realize their self-worth by identifying, capturing, innovating, and creating products and services (Wang and Lv, 2014). Generally, undergraduate entrepreneurship refers to the pioneering activities of undergraduates discovering and taking advantage of opportunities, establishing enterprises based on their knowledge, skills, and novel ideas, making decisions, creating wealth, and realizing their self-worth. This article refers to this definition of undergraduate entrepreneurship.

### Relevant Research on the Entrepreneurial Performance of College Students

There are many factors affecting the entrepreneurial performance of college students. Most of both entrepreneurship (68.9%) and non-entrepreneurship (33.8%) students intend to work for themselves by starting their own businesses (45.2%). Based on this, lack of finance and experience was the most important reason for not working for self, respectively, with 72 and 60.1%. In addition, lack of confidence (51%) and high uncertainty in the market (44.1%) were among the most influential factors for the students (Salamzadeh et al., 2013). Personality traits and a favorable economic environment were proven important in explaining to students the intention to choose an entrepreneurial career as their career choice after they graduated from the study (Awang et al., 2014). Students’ satisfaction with entrepreneurial marketing issues has a great impact on students’ desire to be an entrepreneur (Usman et al., 2013). The profitability of undergraduate entrepreneurship is influenced by various factors. Chen (2020) asserts that the predictive factors of the entrepreneurial performance of college students can be divided into internal and external factors. In addition, Xiong et al. (2021) believes that improvisational behavior can improve entrepreneurial performance under certain conditions. But according to Ma et al. (2021), improvisational behavior in entrepreneurship leads to uncertain outcomes and can even exacerbate problems. So, we will conduct a survey to explore the influence of entrepreneurial improvisational behavior on entrepreneurial performance. This research not only explores the predictive factors of the college students’ entrepreneurial performance at the individual level but also focuses on the level of the college students’ entrepreneurial behavior. Shen and Luo (2006) asserts that financial indicators are the core indicators for measuring the success of a startup. According to the above literature, financial evaluation indicators play a central role in measuring corporate performance. Therefore, this article lists the annual income of enterprises as an effective index to measure the entrepreneurial performance of college students.

## Hypotheses

Entrepreneurship is a complex activity affected by a variety of factors. Prior research has enhanced our understanding of the entrepreneurial performance of undergraduates, and there are some limitations. First, the majority of the studies have focused on the individual factors of college students, neglecting the dynamic effects of the behavioral factors. Second, the research so far has examined either the quantitative form of entrepreneurial performance or qualitative forms of entrepreneurs’ psychological capital, risk propensity, and other personal factors but not both in the same study. It is important to better understand the mechanisms of the relationship between entrepreneurial performance and entrepreneurial traits and behavior from a comprehensive perspective. So, drawing on social cognition theory and strategic adaptation theory, we sought to propose and test a model of how individual factors and behavioral factors affect the performance of new ventures in a sample of entrepreneurs from emerging industries in China. The hypotheses proposed later in this study are mainly based on these two theories.

### Social Cognition Theory

This theory mainly explains the relationship between human social psychology and social behavior, emphasizing that human beings are jointly influenced by the external environment, internal factors, and past and present behaviors (Spencer and Bandura, 1987). People can improve their ability and quality through their own learning. Fundamentally, this theory is a learning theory and is widely used in entrepreneurship research. Entrepreneurship itself is a process of finding opportunities, investing resources, and constantly learning new knowledge and improving their ability to solve various problems. We can recognize the core position of entrepreneurs. Therefore, the research on entrepreneurial performance should attach importance to the measurement of the characteristics of entrepreneurs themselves, and the characteristics and behaviors of entrepreneurial subjects should be added into the framework of the entrepreneurial performance analysis.

### Theory of Strategic Adaptation

This theory focuses on the discovery and utilization of entrepreneurial opportunities and takes it as a starting point, focusing on how the choice of strategy affects entrepreneurial performance in the process of entrepreneurial activities. When research is carried out based on this theory, it is believed that many available opportunities exist in the external environment, and entrepreneurial subjects can develop appropriate strategies to explore and utilize these entrepreneurial opportunities, so the behavioral choices of entrepreneurs will affect entrepreneurial performance. Resources themselves are not dynamic. Only by choosing appropriate strategies can they better serve entrepreneurial activities and achieve better performance (Zehir et al., 2015).

These two theories provide the thinking angle and theoretical support for the following research of the predictive factors of entrepreneurial performance. Social cognition theory reminds us to pay attention to the ability and quality of entrepreneurs, while strategic adaptation theory highlights the importance of behavior selection and direction determination. Based on these two theories, this study analyzes the predictive factors of the entrepreneurial performance of college students based on two levels, namely, the individual and behavioral factors of college students.

### Individual Factors of College Students

Entrepreneurship literacy is necessary for college students to successfully start a business. Entrepreneurship literacy is the inner unity of entrepreneurial knowledge, personality traits, and entrepreneurial ability (Ma, 2020). Following the advent of the entrepreneurial boom, college students are increasingly enthusiastic about entrepreneurship, hoping to prove their value through entrepreneurship. In the start-up period and the mid-stage of a company, the ability of college students affects the survival rate of the company. In the growth stage of entrepreneurial companies, the proportion of the personality traits of entrepreneurs gradually plays a role in the process of entrepreneurship. Therefore, the following hypotheses were made.

H1: The entrepreneurial willingness of college students positively influences their entrepreneurial performance.

H2: The entrepreneurial ability of college students positively influences their entrepreneurial performance.

H3: The personality traits of college students positively influence their entrepreneurial performance.

### Behavioral Factors of College Students

As the main body of entrepreneurial practice, the behavioral factors of college students distinctly contribute to improving the entrepreneurial performance of an enterprise. There are also some studies in Slovakia. Graduate Practice is in Slovakia one of the measures of active labor market policy aimed at decreasing the unemployment rate of young graduates. The specific objective of this intervention is to provide young jobseekers the first contact with the labor market, first work experience, and work habits that may be attractive to potential employers (Lucia et al., 2021). Lucia and Katarína (2021) indicates that the graduate practice had a positive impact on the employability and sustainability of its participants. The impact of behavioral practices on entrepreneurs cannot be underestimated. Entrepreneurial opportunities often originate from market needs that have not been developed or met. New ventures can identify and grasp entrepreneurial opportunities to meet market needs, hence improving corporate performance. Entrepreneurial opportunities have potential value attributes (Guo and Shen, 2014). Some scholars assert that the adventurous and narcissistic tendencies of entrepreneurs have a significant and positive impact on the performance of startups. This may be because, during the start-up period, entrepreneurs’ adventurous tendencies contribute to their discovery of entrepreneurial opportunities (Wang, 2020). Additionally, entrepreneurs’ bold decision-making behavior has a positive impact on the improvement of entrepreneurial performance. When faced with resource shortages and fleeting entrepreneurial opportunities, if entrepreneurs do not have sufficient resources and previous relevant experience, they are most likely to make decisions based on improvisation (Zheng and Long, 2012).

H4: The opportunity-capture behavior of college students positively influences their entrepreneurial performance.

H5: The bold decision-making behavior of college students has a positive impact on their entrepreneurial performance.

## Materials and Methods

### Participants

This study used college students with entrepreneurial experience as the survey subjects. A total of 2,200 questionnaires were distributed to entrepreneurial college students in various universities in Beijing, and 2,097 valid questionnaires were returned. As shown in Table 1, according to gender, the male and female samples accounted for 81.4 and 18.6%, respectively. Samples from research and ordinary universities account for 35.7 and 64.3%, respectively; 33.3%, 61.5%, 4.1%, and only 10% of the enterprises are in the initial, growth, expansion, and mature stages, respectively. This is consistent with the basic situation of college student entrepreneurs, as a young group. Overall, the samples were representative.

**TABLE 1:** Basic descriptive statistics of the questionnaire.

Basic information of college entrepreneurs		Frequency	Percentage
Gender	Male	1706	81.4%
	Female	391	18.6%
School	Research university	749	35.7%
	General colleges	1348	64.3%
Entrepreneurial stage	Early stage	699	33.3%
	Growth stage	1290	61.5%
	Expansion stage	86	4.1%
	Mature stage	22	1.0%

### Procedure

All the tests in this study were filled out by undergraduate entrepreneurs. The professors sent online questionnaires to many undergraduate entrepreneurs who graduated from their colleges. The individual and behavioral factors of college students may influence entrepreneurial performance. This questionnaire was based on these two dimensions by referring to relevant domestic and foreign literature. This research was approved by the Research Ethics Committee of the School of Humanities and Social Sciences, the University of Science and Technology, Beijing.

### Measures

#### Questionnaire Design

The entrepreneurial performance of college student entrepreneurs is affected by many factors. This study mainly examines the degree of the influence of individual and behavioral factors. Therefore, the independent variables in this study are divided into five aspects, namely, entrepreneurial willingness, entrepreneurial ability, personality traits, the art of capturing opportunities, and bold decision-making behavior. The dependent variable is the annual income of entrepreneurial enterprises, which ranges from “below 5,00,000 yuan” and “5,00,000–1 million yuan” to “3.05–5 million yuan” and “over 5 million yuan.” The scale was compiled using a 5-point scale. The questionnaire was designed such that each predictive factor contained five items, namely, “very unimportant” (1 point), “not very important” (2 points), “general” (3 points), “relatively important” (4 points), and “very important” (5 points). Through the analysis, it was established that the questionnaire had good reliability and validity. A total of 2,200 questionnaires were then distributed. The questionnaires were further collected and selected after which, 2,097 valid questionnaires were obtained.

#### Reliability and Validity of the Questionnaire Sample

This study used the MPLUS program to calculate the reliability of the overall scale and subscale and used Cronbach’s coefficient value. Table 2 shows the results. The overall consistency coefficient of the questionnaire sample was 0.900 > 0.8, indicating that the overall reliability of the questionnaire sample was relatively good. All the Cronbach’s coefficient values of the scale were above 0.6. Subsequently, the validity of the scale was analyzed. Table 3 shows that the overall Kaiser–Meyer–Olkin (KMO) value of the scale is 0.865 and that the results of the Bartley sphere test are significant. Therefore, this scale has good validity.

**TABLE 2:** Reliability test results.

Variable	Item	Cronbach’s alpha	Cronbach’s alpha of item deleted
Personal personality traits	Perseverance	0.884	0.895
	Social responsibility		0.894
	Honesty		0.894
	Tolerance		0.893
	Leadership charm		0.892
	Mutual benefit		0.892
	Confident		0.894
Personal entrepreneurial ability	Creativity	0.882	0.896
	Management ability		0.892
	Foreign cooperation ability		0.893
	Market development ability		0.893
	Product development ability		0.892
Opportunity capture behavior	Dare to take risks	0.763	0.895
	Funding status		0.896
	Risk control status		0.893
Bold decision behavior	Opportunity recognition status	0.750	0.894
	Team formation status		0.894
	Project learning status		0.894
	Entrepreneurship project is fully feasible		0.897
	Dare to take responsibility		0.895
Personal entrepreneurial willingness	Entrepreneurship is an effective means to prove one’s own ability	0.676	0.903
	Entrepreneurship can help one realize their self-worth		0.899
	Have great entrepreneurial enthusiasm		0.898

Total		0.900	

**TABLE 3:** Validity test results.

Category	KMO value	Bartlett test of Sphericity		
		Chi-square test	Degree of freedom	Significance
Personal personality traits	0.886	8501.774	21	0
Personal entrepreneurial ability	0.854	5364.348	10	0
Opportunity capture behavior	0.673	1706.700	3	0
Bold decision behavior	0.819	3082.654	10	0
Personal entrepreneurial willingness	0.596	1145.621	3	0

Total	0.865	31719.807	275	0

#### Variable Interpretation

This study refers to personal entrepreneurial willingness as the intensity of the intention of college student entrepreneurs to engage in entrepreneurship, including whether they have great entrepreneurial enthusiasm and whether they believe that entrepreneurship can help them realize their self-worth and prove their capabilities.

Personal entrepreneurial ability refers to the ability of college student entrepreneurs to successfully establish and persistently operate a business; this also encompasses innovation, management, external cooperation, market development, and product development abilities.

Personal personality traits refer to the personality traits including persistence, honesty, self-confidence, tolerance, leadership charm, social responsibility, and mutual benefit that college student entrepreneurs should possess as founders and leaders of enterprises.

Bold decision-making behavior refers to the ability of entrepreneurs to make decisive decisions after considering factors such as entrepreneurial project preparation, team formation, project learning, clear opportunities, and risk-taking responsibility.

The behavior of capturing opportunities refers to the art of college student entrepreneurs to take risks and seize market opportunities after assessing the status of fund acquisition and risk control.

This study lists the annual income of enterprises as an effective indicator to measure the entrepreneurial performance of college students. The financial evaluation indicators play a central role in measuring the entrepreneurial performance of an enterprise.

#### Model Construction

The structural equation model is a multivariate statistical method. To analyze the predictive factors of the entrepreneurial performance of college students, relevant “latent variables” should be set here to describe the corresponding indicators extracted from the questionnaire. There are six latent variables, namely, personal entrepreneurial willingness, personal entrepreneurial ability, personal personality traits, the art of capturing opportunities, and bold decision-making behavior, and 23 observed variables; the entrepreneurial performance of college students is measured using “enterprise annual income.” A complete structural equation model is further constructed using the above parameters.

## Results

### Structural Equation Model

This study used the MPLUS program to analyze the relationship between the entrepreneurial performance of college students and entrepreneurial willingness, entrepreneurial ability, personality traits, the art of capturing opportunities, and bold decision-making behavior. The figure below shows the structural equation model. Goodness-of-fit index (GFI), comparative fit index (CFI), and root mean square error of approximation (RMSEA) are the three indicators used to verify the plan. The data of the fitness test are as follows: GFI = 0.975, CFI = 0.981, and RMSEA = 0.060. According to the index values above, the measurement model of this study has a high degree of fit with the actual data of the questionnaire survey. The model also exhibits a good fit.

### Path Test of Direct Effects

Table 4 shows that the personal entrepreneurial ability, personal personality traits, art of capturing opportunities, and bold decision-making behavior were significant at a *P* < 0.05 level. However, personal entrepreneurial willingness was insignificant. The test results mean that in the entrepreneurs’ perception, personal entrepreneurial willingness insignificantly influences the entrepreneurial performance of college students. Hypothesis H1 is invalid; the personal entrepreneurial ability has a significant positive effect on the entrepreneurial performance of college students (β = 0.868, *P* < 0.01). Hypothesis H2 is supported by data; personal personality traits have a significant positive impact on the entrepreneurial performance of college students (β = 0.288, *P* < 0.05). Hypothesis H3 is supported by data; the art of capturing opportunities has a significant effect on the entrepreneurial performance of college students (β = 0.357, *P* < 0.05). Hypothesis H4 is supported by data; bold decision-making behavior has a significant positive impact on the entrepreneurial performance of college students (β = 0.312, *P* < 0.05), it is assumed that hypothesis H5 is supported by data.

**TABLE 4:** Research hypotheses testing.

Path	Standardized path coefficient	S.E.	*Z* (CR value)	*P*	Correspondence hypothesis	Significance
Personal entrepreneurial willingness → The entrepreneurial performance of college students	−0.027	0.157	−0.695	0.487	H1	Insignificant
Personal entrepreneurial ability → The entrepreneurial performance of college students	0.868	0.547	5.822	0.000	H2	Significant
Personal personality traits → The entrepreneurial performance of college students	0.288	0.315	3.120	0.002	H3	Significant
The behavior of capturing opportunities → The entrepreneurial performance of college students	0.357	0.316	3.051	0.002	H4	Significant
Bold decision-making behavior → The entrepreneurial performance of college students	0.312	0.383	3.218	0.001	H5	Significant

## Discussion

### Personal Entrepreneurship Ability and the Entrepreneurial Performance of Undergraduates

**FIGURE 1:**
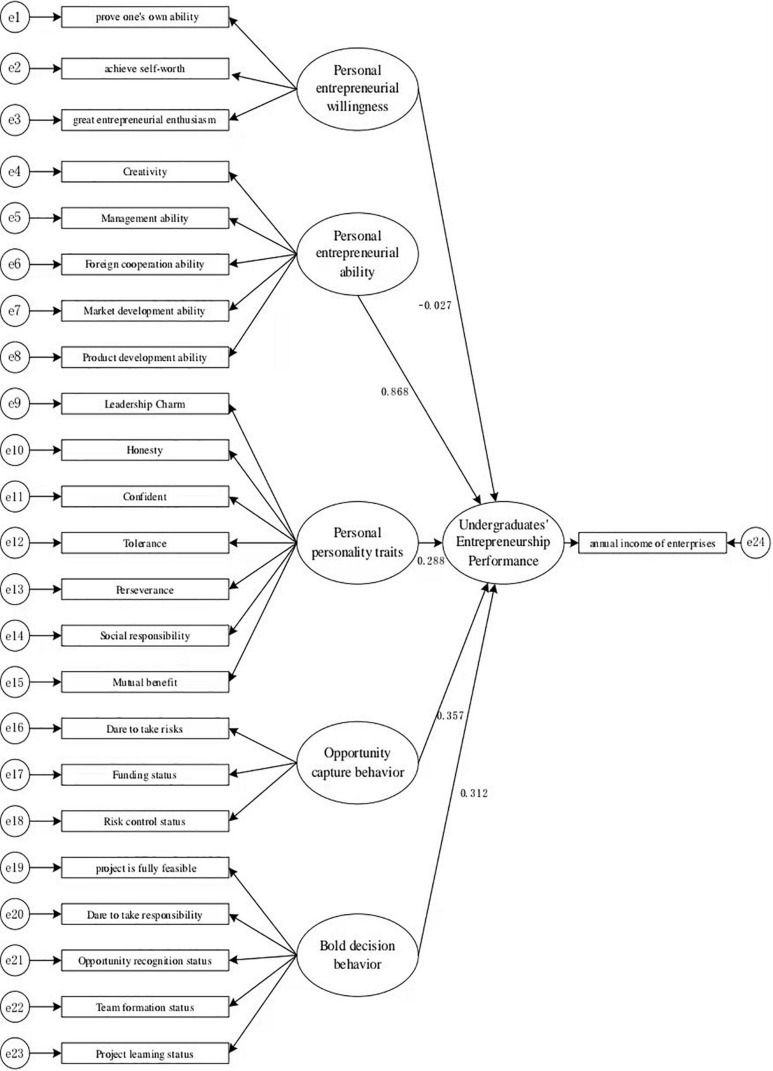
Structural equation model.

By analyzing the structural equation model in Figure 1, it is clear that “personal entrepreneurial ability” has the greatest impact on the entrepreneurial performance of college students, accounting for a standardized path coefficient value of 0.868. Among these abilities, market development and management abilities play particularly prominent roles, with coefficient values of 0.820 and 0.817, respectively. Most startups are striving to open up unoccupied market sites. In this case, startups need to capture market shares as quickly as possible. Effective market development can generate new performance growth points for enterprises. The management ability of entrepreneurs is also crucial to improving corporate performance. In the era of the knowledge economy, the level of talent management ability of enterprise managers directly affects the competitiveness of enterprises (Xia and Liu, 2015). Additionally, the speed of the introduction of new products into the market significantly influences the performance of new startups (Kong et al., 2013). Enterprise innovation can also promote enterprise performance. The positive effect of technological innovation on the performance of an enterprise is particularly obvious in the performance of high-tech enterprises (Zhang et al., 2021). However, the impact of technological innovation is lagging and must be combined with scientific company management. The external cooperation and communication capabilities of entrepreneurs are also crucial for the continuous growth of corporate performance. The excellent talent outreach capabilities of entrepreneurs enable them to seek opportunities, attract investment in external exchanges and cooperation, and help startup companies to grow steadily (Huang and Liang, 2021). The improvement of entrepreneurial performance is inseparable from the leadership of excellent entrepreneurs. Entrepreneurs with self-management and self-supervision capabilities have more opportunities to accumulate knowledge and experience during entrepreneurial development (Chien-Chi, 2013). College student entrepreneurs should consciously improve themselves and cultivate their entrepreneurial abilities and personality.

### Personal Personality Traits and the Entrepreneurial Performance of Undergraduates

The personality traits of undergraduate entrepreneurs also influence corporate performance. The model shows that leadership charm has the greatest impact, with a coefficient value of 0.873. Entrepreneurs’ spiritual leadership can improve entrepreneurial performance (Ge and Gu, 2016). The concept of spiritual leadership of entrepreneurs was first proposed by Louis (2003). Spiritual leadership refers to the ability of entrepreneurs to use their leadership charm to stimulate their own and other people’s sense of intrinsic value realization and spiritual wellbeing. Additionally, the reciprocal altruistic character and sense of social responsibility of college student entrepreneurs help to increase the loyalty of employees to the entrepreneurial enterprise and contribute more to the realization of the long-term economic benefits of the enterprise (Cao, 2009). In this study, self-confidence indicates moderate self-confidence. Research shows that overconfident management has a negative impact on corporate performance (Xia and Dong, 2019). In conclusion, setbacks are inevitable during the process of entrepreneurship. However, a great personal personality allows entrepreneurs to handle difficulties more calmly, enabling startups to operate for a long period of time. Good entrepreneurial education will positively influence the future entrepreneurial performance of college student entrepreneurs (Mercy et al., 2018). Universities should also offer not only conventional business courses (e.g., business planning) but also new forms of education so that students meet various entrepreneurial tasks and problems, make decisions in different situations, explore and acquaint themselves with entrepreneurship, make practical curriculum reforms, and pay more attention to entrepreneurial education and students’ personality (Gubik and Farkas, 2016).

### Personal Entrepreneurial Willingness and the Entrepreneurial Performance of Undergraduates

By analyzing the structural equation model data, it is clear that the personal entrepreneurial willingness of college students insignificantly influences entrepreneurial performance. For the process-based motives, perceived feasibility and perceived desirability to start a social enterprise, as well as exposure to entrepreneurship, are significant predictors of students’ intention to form a social enterprise (Barton et al., 2018). Perceived behavioral control and subjective norm were proved as significant mediators in individual entrepreneurial orientation and entrepreneurial intention relationships (Awang et al., 2016). Entrepreneurship is a complex and risky activity, where goals are not easily achieved. In the present entrepreneurial behavior of college students, personal entrepreneurial willingness plays a limited role in improving corporate performance, and it is necessary to have sufficient ability and determination to truly contribute to the development of entrepreneurship.

### Art of Capturing Opportunities and the Entrepreneurial Performance of Undergraduates

This study sets up two dimensions at the level of entrepreneurial behavior: “the art of capturing opportunities” and “bold decision-making behavior.” These two dimensions had a similar impact on the entrepreneurial performance of college students with coefficient values of 0.357 and 0.312, respectively. A proactive personality positively and significantly moderated the relationship between entrepreneurial intention and entrepreneurial behavior (Li et al., 2020). In the present market, opportunities are fleeting, and there are many competitors in the entrepreneurial field. If entrepreneurs hesitate, they can easily lose opportunities. Capital turnover and risk assessment require a lot of time, which is negatively related to the art of the timely capturing of opportunities. Therefore, startup companies must take the lead in identifying and seizing opportunities, using fresh ideas to invent new products that can generate profits through research, and opening up markets to promote growth in corporate performance (Ma, 2019). A good institutional environment has a positive regulatory effect on the performance of entrepreneurial enterprises (Yu, 2013). Government support has a significant positive impact on the survival, performance, and improvement of entrepreneurial enterprises (Xiang and Sun, 2021). The government can create a friendly public environment and system for college student entrepreneurs.

### Bold Decision-Making Behavior and the Entrepreneurial Performance of Undergraduates

Analyzing the “bold decision-making behavior” at the level of entrepreneurial behavior shows that entrepreneurs should be responsible for making bold decisions with basic certainty instead of hesitating to consider the pros and cons of opportunities, team building, and project learning details in a long time. If entrepreneurs are conservative in the face of risks and do not make bold decisions, they will easily fall into the quagmire of the conservative ways and consequently, miss the “breakthrough” opportunities of entrepreneurial enterprises. Entrepreneurship involves numerous risks and uncertainties. Positive psychological resources such as courage, as well as confidence, hope, optimism, and resilience, can be valuable for entrepreneurs (Bockorny and Youssef-Morgan, 2019). The research found that entrepreneurial education had direct effects on entrepreneurial behavior and psychological capital (Cui, 2021). The developmental quality of entrepreneurial tasks had a positive relationship with venture performance, and this relationship was mediated by entrepreneurial action learning (Chen and Pan, 2019). Universities need to provide students with training opportunities and carry out high-quality entrepreneurship education such as improving the external environment of entrepreneurship, improving the entrepreneurship curriculum in higher education institutions, improving the teaching staff, and developing the practice bases for entrepreneurship education (An and Xu, 2021), so as to enhance students’ courage, confidence, and decision-making judgment.

## Conclusion

College students are the main forces in entrepreneurship. The number of young entrepreneurs devoted to entrepreneurship is increasing in recent years. In contrast to this great momentum, many problems that urgently need to be studied and solved have emerged. The unsatisfactory performance of undergraduates in entrepreneurship is one of the problems worthy of studying. Through research and analysis, this study finds that the entrepreneurial performance of college students is affected by many factors at the individual and behavioral levels. Their personality traits, entrepreneurial abilities, the art of capturing opportunities, and bold decision-making behavior influence the performance of entrepreneurial companies. Through various efforts, the entrepreneurial performance of undergraduate entrepreneurs will gradually improve, and China’s economy will surely experience a longer-term development.

## Data Availability Statement

The original contributions presented in the study are included in the article/Supplementary Material, further inquiries can be directed to the corresponding author.

## Ethics Statement

The studies involving human participants were reviewed and approved by University of Science and Technology Beijing. Written informed consent to participate in this study was provided by the participants.

## Author Contributions

QS contributed to the construction of the overall framework of the manuscript. LT was responsible for the analysis and processing of the data. ZM was responsible for the writing of the manuscript. All authors contributed to the article and approved the submitted version.

## Conflict of Interest

The authors declare that the research was conducted in the absence of any commercial or financial relationships that could be construed as a potential conflict of interest.

## Publisher’s Note

All claims expressed in this article are solely those of the authors and do not necessarily represent those of their affiliated organizations, or those of the publisher, the editors and the reviewers. Any product that may be evaluated in this article, or claim that may be made by its manufacturer, is not guaranteed or endorsed by the publisher.

## References

[B1] An H. J. Xu Y. Y. (2021). Cultivation of entrepreneurial talents through virtual entrepreneurship practice in higher education institutions. *Front. Psychol.* 12:690692. 10.3389/FPSYG.2021.690692 34393916 PMC8357997

[B2] Awang A. Amran S. Nor M. N. M. Ibrahim I. I. Razali M. F. M. (2016). Individual entrepreneurial orientation impact on entrepreneurial intention: intervening effect of PBC and subjective norm. *J. Entrepreneurship Bus. Econ.* 4 94–129.

[B3] Awang A. Ibrahim I. I. Ayub S. A. (2014). Determinants of entrepreneurial career: experience of polytechnic students. *J. Entrepreneurship Bus. Econ.* 2 21–40.

[B4] Barton M. Schaefer R. Canavati S. (2018). To be or not to be a social entrepreneur: motivational drivers amongst American business students. *Entrepreneurial Bus. Econ. Rev.* 6 9–35. 10.15678/eber.2018.060101

[B5] Bockorny K. Youssef-Morgan C. M. (2019). Entrepreneurs’ courage, psychological capital, and life satisfaction. *Front. Psychol.* 10:789. 10.3389/fpsyg.2019.00789 31024410 PMC6461011

[B6] Cao H. M. (2009). Research on corporate performance evaluation based on social responsibility. *Econ. Problems* 12 60–62. 10.16011/j.cnki.jjwt.2009.12.018

[B7] Chen Y. N. (2020). A preliminary study on the improvement path of college students’ entrepreneurial performance. *Industrial Technol. Innov.* 17 123–124.

[B8] Chen Y. N. Pan J. Y. (2019). Do entrepreneurs’ developmental job challenges enhance venture performance in emerging industries? a mediated moderation model of entrepreneurial action learning and entrepreneurial experience. *Front. Psychol.* 10:1371. 10.3389/fpsyg.2019.01371 31244744 PMC6579820

[B9] Chien-Chi T. (2013). Connecting self-directed learning with entrepreneurial learning to entrepreneurial performance. *Int. J. Entrepreneurial Behav. Res.* 19 425–446.

[B10] Cui J. (2021). The influence of entrepreneurial education and psychological capital on entrepreneurial behavior among college students. *Front. Psychol.* 12:755479. 10.3389/FPSYG.2021.755479 34867651 PMC8638359

[B11] Ge Y. Gu J. P. (2016). Entrepreneur’s spiritual leadership, strategic flexibility and entrepreneurial performance: the moderating role of environmental dynamics. *Modernization Manag.* 76–78. 10.3969/j.issn.1003-1154.2016.03.023

[B12] Gubik A. S. Farkas S. (2016). Student entrepreneurship in hungary: selected results based on guesss survey. *Entrepreneurial Bus. Econ. Rev.* 4:123. 10.15678/eber.2016.040408

[B13] Guo H. Shen R. (2014). How to transform entrepreneurial opportunities into corporate performance: the mediating role of business model innovation and the moderating role of market environment. *Econ. Theory Bus. Manage.* 34 70–83. 10.3969/j.issn.1000-596X.2014.03.007

[B14] Huang R. G. Liang H. W. (2021). Research on the influence of talent outreach ability on innovation and entrepreneurship performance——based on the perspective of social network connection. *Asia Pac. Econ. Rev.* 37 123–128. 10.16407/j.cnki.1000-6052.20210326.014

[B15] Kong T. Sun L. Y. Feng T. W. (2013). Research on the relationship between customer orientation, new product launch speed and corporate performance. *Nankai Bus. Rev.* 16 90–99. 10.3969/j.issn.1008-3448.2013.05.010

[B16] Li C. Murad M. Shahzad F. Khan M. A. S. Ashraf S. F. Dogbe C. S. K. (2020). Entrepreneurial passion to entrepreneurial behavior: role of entrepreneurial alertness, entrepreneurial self-efficacy and proactive personality. *Front. Psychol.* 11:1611. 10.3389/fpsyg.2020.01611 32973593 PMC7468520

[B17] Louis W. F. (2003). Toward a theory of spiritual leadership. *Leadership Quarterly* 14 693–727. 10.1016/j.leaqua.2003.09.001

[B18] Lucia Š Katarína K. (2021). An analysis of participation factors and effects of the active labour market measure Graduate practice in Slovakia – Counterfactual approach. *Eval. Program Plann.* 86:101917. 10.1016/j.evalprogplan.2021.101917 33611162

[B19] Lucia Š Katarína K. Marek Ï (2021). Evaluation of the effects of the graduate practice in slovakia: comparison of results of counterfactual methods. *Central Eur. Bus. Rev.* 10 1–31. 10.18267/j.cebr.266

[B20] Ma H. J. Wu J. Guo H. Ge B. S. (2021). Improvisation in entrepreneurship: antecedents, consequences, and boundary conditions. *Manage. World* 37 211–227. 10.19744/j.cnki.11-1235/f.2021.0074

[B21] Ma L. (2019). The impact of new ventures’ innovation drive and entrepreneurial ability of new ventures on corporate growth performance: a mediating role. *Enterp. Econ.* 39 49–59. 10.13529/j.cnki.enterprise.economy.2019.09.006

[B22] Ma S. H. (2020). Entrepreneurship literacy: the necessary literacy for college students to successfully start a business. *J. Innov. Enterprise Educ.* 11 32–37. 10.3969/j.issn.1674-893X.2020.01.007

[B23] Mercy E. O. Maxwell A. O. John U. Chinonye L. M. (2018). Data On entrepreneurship education and entrepreneurial performance of aspiring entrepreneurs in selected nigerian universities. *Data Brief* 20 108–112. 10.1016/j.dib.2018.07.044 30109247 PMC6089197

[B24] Salamzadeh A. Azimi M. A. Kirby D. A. (2013). Social entrepreneurship education in higher education: insights from a developing country. *Int. J. Entrepreneurship Small Bus.* 20 17–34. 10.1504/ijesb.2013.055691 35009967

[B25] Shen C. H. Luo L. (2006). Research on the key factors of entrepreneurship success and entrepreneurship performance indicators. *J. Central South University (Social Science Edition)* 12 231–235. 10.3969/j.issn.1008-7621.2021.02.011

[B26] Spencer E. C. Bandura A. (1987). Social foundations of thought and action: a social cognitive theory. *Contemp. Sociol.* 16 12–13. 10.2307/2071177

[B27] Usman Y. Altaf M. Rani Z. Alam M. Aslam M. (2013). Improving entrepreneural marketing learning: a study of business graduates, Pakistan. *J. Womens Entrep. Educ.* 56 74–89.

[B28] Wang L. J. Lv J. Y. (2014). Study on the Chinese path of learning and borrowing Schumpeter’s innovation and entrepreneurship thoughts. *Jiangsu Soc. Sci.* 35 267–271. 10.13858/j.cnki.cn32-1312/c.2014.06.037

[B29] Wang X. X. (2020). *Research on the Influence of Entrepreneur’s Characteristics on the Performance of Entrepreneurial Enterprises.* China: Xi’an Shiyou University.

[B30] Xia G. X. Dong S. (2019). The relationship between internal control, manager overconfidence and corporate performance. *Friends Account.* 19 120–125. 10.3969/j.issn.1004-5937.2019.20.020

[B31] Xia J. H. Liu D. R. (2015). Enhancing the knowledge management ability of the management of science and technology enterprises. *Soc. Sci.* 16 78–82. 10.3969/j.issn.1002-3240.2015.01.014

[B32] Xiang S. H. Sun Y. H. (2021). Research on the influence mechanism of government support on the entrepreneurial performance of high-level talents. *Sci. Technol. Prog. Policy* 38 143–150. 10.6049/kjjbydc.2020120228

[B33] Xiong L. Jia J. F. Zhu J. B. Liu B. (2021). *What Triggers Improvisation? Sociological Investigation Based on Entrepreneur Behavior Nankai Business Review.* Available online at: https://kns.cnki.net/kcms/detail/12.1288.F.20210909.1435.008.html (accessed September 10, 2021).

[B34] Yu X. Y. (2013). Network ability, technical ability, institutional environment and international entrepreneurial performance. *J. Manag. Sci.* 26 13–27. 10.3969/j.issn.1672-0334.2013.02.002

[B35] Zehir C. Can E. Karaboga T. (2015). Linking entrepreneurial orientationto firm performance: the roleof differentiation strategy and innovation performance. *Proc. Soc. Behav. Sci.* 210 358–367. 10.1016/j.sbspro.2015.11.381

[B36] Zhang W. D. Cui C. J. Wang Z. (2021). Research on the relationship between technological innovation and corporate performance based on the regulatory effects of governance mechanisms: empirical data from listed high-tech companies. *Statistics Inform. Forum* 36 107–118. 10.3969/j.issn.1007-3116.2021.03.009

[B37] Zheng X. Z. Long D. (2012). Review and prospects of entrepreneurial decision-making research based on the process view. *Foreign Econ. Manag.* 34 11–17. 10.16538/j.cnki.fem.2012.08.007

