# Supplementary material for: Predictive Factors of the Entrepreneurial Performance of Undergraduates
Source: Front Psychol. 2022 Mar 11;13:814759. doi: 10.3389/fpsyg.2022.814759 (PMC8963528; doi:10.3389/fpsyg.2022.814759)
Supplement: Supplementary file 1 [file Data_Sheet_1.docx]

**Appendix**

**Survey Questionnaire**

Dear Sirs：

Hello! In order to fully understand the current situation of entrepreneurship performance of college students in China, we have designed this questionnaire and kindly ask you to help fill it out.

The purpose of this questionnaire is to know the entrepreneurial process and the personality of the entrepreneur. It does not involve the evaluation of your company's work, nor is it used for any ranking. The feedback information of the questionnaire will be processed anonymously and then recorded into the computer for statistical analysis. Please feel free to fill in the questionnaire according to the real situation of your company and your true thoughts.

Requirements:

1. Please let the principal founder of your company fill in this form.

2. Please tick √ or insert the appropriate words or numbers in the box.

Thank you for your support!

**Basic personal information**

Q1.Gender：(1) □Male (2)□Female

Q2. What degree did you have when you started your first business?

(01)□Junior college and below (02)□Bachelor degree (03)□Master Degree (04)□Doctoral degree

Q3. What kind of university did you attend?

(01) □Research university (02)□General colleges

Q4. What stage is your business in?

(01)□Early stage (02)□Growth stage (03) □Expansion stage (04)□Mature stage

（1. Early stage：The enterprise has been founded for a short time, and there is no finalized product. It is still in the state of primitive accumulation and market exploration 2. Growth stage：The enterprise runs stably, already has main product 3. Expansion stage：The enterprise is expanding marketing and improving their competitiveness step by step 4. Mature stage：Enterprise products, technology, management system has been mature, product sales scale ）

**Evaluation of entrepreneur characteristics**

|  | Strongly agree | relatively agree | general | not very agree | strongly disagree |
| --- | --- | --- | --- | --- | --- |
| **A. Personal entrepreneurial willingness** | | | | | |
| A-1 Have great entrepreneurial enthusiasm | □ | □ | □ | □ | □ |
| A-2 Entrepreneurship can help one realize their self-worth | □ | □ | □ | □ | □ |
| A-3 Entrepreneurship is an effective means to prove one's own ability | □ | □ | □ | □ | □ |
| **B.** **Personal personality traits（Please choose the personality traits that stand out in your own business.** **Choose a maximum of 4 "very important" options）** | very important | relatively important | general | not very important | very unimportant |
| B-1 Leadership Charm | □ | □ | □ | □ | □ |
| B-2 Honesty | □ | □ | □ | □ | □ |
| B-3 Confident | □ | □ | □ | □ | □ |
| B-4 Tolerance | □ | □ | □ | □ | □ |
| B-5 Perseverance | □ | □ | □ | □ | □ |
| B-6 Social responsibility | □ | □ | □ | □ | □ |
| B-7 Mutual benefit | □ | □ | □ | □ | □ |
| **C.** **Personal entrepreneurial ability（Please choose the personality entrepreneurial ability that stand out in your own business. Choose a maximum of 4 "very important" options）** | very important | relatively important | general | not very important | very unimportant |
| C-1 Creativity | □ | □ | □ | □ | □ |
| C-2 Management ability | □ | □ | □ | □ | □ |
| C-3 Foreign cooperation ability | □ | □ | □ | □ | □ |
| C-4 Market development ability | □ | □ | □ | □ | □ |
| C-5 Product development ability | □ | □ | □ | □ | □ |
| **D.** **Opportunity capture behavior** | very important | relatively important | general | not very important | very unimportant |
| D-1 Dare to take risks | □ | □ | □ | □ | □ |
| D-2 Funding status | □ | □ | □ | □ | □ |
| D-3 Risk control status | □ | □ | □ | □ | □ |
| **E.** **Bold decision behavior** | very important | relatively important | general | not very important | very unimportant |
| E-1 Dare to take responsibility | □ | □ | □ | □ | □ |
| E-2 Opportunity recognition status | □ | □ | □ | □ | □ |
| E-3 Team formation status | □ | □ | □ | □ | □ |
| E-4 Project learning status | □ | □ | □ | □ | □ |

**Entrepreneurial performance**

Q1. How long has the enterprise been in operation?

(01)□Within 1 year (02)□1-3years (03)□3-5years (04)□More than 5 years

Q2. What is the current profit status of your enterprise?

(01)□Profit a lot (02)□Profit in general (03)□Break even （4)□At a loss

Q3. If profitable, your annual gross profit is:?

(01)□Less than 500,000 yuan (02)□510,000 yuan -1 million yuan (03)□1.01 million -3 million (04)□3.01 million yuan to 5 million yuan (05)□More than 5 million yuan
